# Opportunities for Telemedicine to Improve Parents’ Well-Being During the Neonatal Care Journey: Scoping Review

**DOI:** 10.2196/60610

**Published:** 2024-12-02

**Authors:** Josephine Wagenaar, Crystal Mah, Fredrik Bodell, Irwin Reiss, Maaike Kleinsmann, Sylvia Obermann-Borst, H Rob Taal

**Affiliations:** 1 Department of Neonatal and Pediatric Intensive Care, Sophia Childrens’ Hospital, Erasmus Medical Center, Wytemaweg 80, Rotterdam, 3015CN, Netherlands, 31 107040704; 2 Department of Design Organization and Strategy, Faculty of Industrial Design Engineering, Delft University of Technology, Delft, Netherlands; 3 Care4Neo, Rotterdam, Netherlands

**Keywords:** telemedicine, telehealth, neonatal care, parental well-being, family-centered care, pediatrics, neonates, newborns, parents, neonatal intensive care unit, NICU

## Abstract

**Background:**

Neonatal intensive care unit admissions of newborns are emotional and stressful for parents, influencing their mental and physical well-being and resulting in high rates of psychological morbidities. Significant research has been undertaken to understand and quantify the burden of a newborn’s medical journey on parents’ well-being. Simultaneously, an increase has been observed in the development and implementation of telemedicine interventions, defined as the remote delivery of health care. Telemedicine is used as an overarching term for different technological interventions grouped as real-time audio-visual communication, remote patient monitoring, and asynchronous communication. Various telemedicine interventions have been proposed and developed but scarcely with the primary goal of improving parental well-being during their newborn’s medical journey.

**Objective:**

This study aims to identify telemedicine interventions with the potential to improve parents’ well-being and to present the methods used to measure their experience.

**Methods:**

A scoping review was conducted, including empirical studies evaluating telemedicine in neonatal care that either measured parental well-being or included parents in the evaluation. Abstract and title screening, full-text screening, and data extraction were performed by three researchers. Two researchers were needed to reach decisions on both the inclusion and extraction of articles.

**Results:**

The review included 50 out of 737 screened articles. Telemedicine interventions focused mainly on daily visits at the neonatal intensive care unit and discharge preparedness for parents. Surveys were the primary tool used for outcome measurement (36/50, 72%). Aspects of parents’ well-being were evaluated in 62% (31/50) of studies. Telemedicine interventions developed to provide education and support showed a potential to improve self-efficacy and discharge preparedness and decrease anxiety and stress when they included a real-time telemedicine component.

**Conclusions:**

This scoping review identified specific telemedicine interventions, such as real-time audio-visual communication and eHealth apps, that have the potential to improve parental well-being by enhancing self-efficacy and discharge preparedness, and reducing anxiety and stress. However, more insights are needed to understand how these interventions affect well-being. Parents should be included in future research in both the development and evaluation stages. It is important to not only measure parents’ perceptions but also focus on the impact of a telemedicine intervention on their well-being.

## Introduction

Admission of a newborn to a neonatal intensive care unit (NICU) places a high emotional burden on parents [1]. This often unexpected neonatal admission exposes the parents to a high risk of developing psychological morbidities, including posttraumatic stress disorder [2]. Parental well-being, referring to the overall mental, emotional, and physical health of parents during and directly after the medical journey of their newborn, influences child development [3,4] and the risk of developing long-term psychological morbidities for the parents [5]. Their well-being is influenced by the stress or anxiety they experience due to the severity of the child’s medical condition [6,7]. Additionally, their mechanisms for coping with the medical situation and traumatic moments, as well as their self-efficacy and confidence in their parenting skills, can further influence their well-being [7-9].

Despite the increasing awareness of the relevance of parents’ well-being during the neonatal care journey, insights into parents’ needs and effective interventions to enhance their well-being are lacking [10]. Steps have been taken to identify needs and factors that influence parental well-being [10-13]. Identified parental needs during the care journey include informational needs, emotional needs, involvement in decision-making, financial needs, practical needs, and ways to cope with transfers and discharge [10,11,14]. Furthermore, parent-infant bonding and social support are important factors associated with depressive symptoms within the first 12 months after discharge from the NICU [12,15]. Discharge from the hospital is affected by communication, unmet informational needs, and the management of expectations and perceptions, exposing an important role for peer support and improved communication by health care providers [13]. Consequently, suggested opportunities to improve parental well-being often focus on communication and informational provisions [16].

Telemedicine interventions are emerging, including within neonatal care [17]. Telemedicine is defined as the remote delivery of health care [18] and is often grouped in (1) remote patient monitoring, (2) real-time health care provider–to–health care provider or health care provider–to–patient consultations, and (3) asynchronous (non–real-time) telecommunication [19]. With telemedicine interventions focusing on the transfer of information, communication, and participation by family members, it has the potential to fulfill the exposed parental needs described above [20]. The importance of parental involvement when developing, evaluating, and implementing telemedicine interventions is underscored by the noticeable increase in the use of patient/parent-reported experience measures (PREMs) and patient/parent-reported outcome measures (PROMs) for evaluating telemedicine interventions [21]. Despite the potential and growing use of PREMs and PROMs, telemedicine interventions are rarely implemented with the primary aim of enhancing parental well-being [22,23]. Therefore, this study aimed to identify telemedicine interventions that potentially enhance parental well-being during the neonatal care journey by performing a scoping review.

## Methods

The scoping review was conducted following the PRISMA-ScR (Preferred Reporting Items for Systematic Reviews and Meta-Analyses Extension for Scoping Reviews) guidelines for scoping reviews [24].

### Eligibility Criteria

Using the PICO (Population, Intervention, Comparator, Outcome) framework, the following inclusion criteria were defined:

- The population/setting of the study must be during the neonatal care journey.
- The intervention must be a telemedicine intervention.
- There was no restriction on the comparator group.
- The outcome measures should include aspects of parental well-being or parents’ perception of the intervention.
- The included studies must be published and use an empirical study design.

The neonatal care journey was demarcated from the neonate’s admission to a neonatal ward in the first week of life and ranged from immediately after birth to 12 months of follow-up. Therefore, studies were excluded when they included only healthy neonates, neonates admitted to a nursery directly after birth, or neonates with congenital abnormalities admitted to a hospital ward later than the first week of life. Telemedicine was defined as the remote delivery of health care, without any restrictions on the technologies used [18]. Long existing technologies, such as telephonic consultations or SMS updates, were purposefully included in this review to include the whole variety of interventions. However, digital health applications that did not provide health care were excluded, such as electronic patient record systems, medical decision support tools, organization tools, or interventions used for training medical professionals. Eligible outcome measures including parental well-being or parents’ perception of the intervention were (1) PREMs, for instance focusing on the usability or satisfaction of the intervention; (2) measures on how the intervention influences the parent’s journey, for instance, travel time saved; and (3) PROMs regarding their well-being, such as depression, anxiety, self-efficacy, or posttraumatic stress disorder. These outcome measures were not confined to quantitative outcomes or validated questionnaires, as qualitative studies can provide a more nuanced description of experiences.

Articles published before the year 2000, not available in English or Dutch, without full-text availability, or with a nonempirical study design (protocols, reviews, editorials, etc) were excluded.

### Search Strategy

Medline, Embase, Web of Science, Cochrane, and Google Scholar databases were searched using the following search terms: telemedicine, neonatal care, parents or caregivers, and experience or perspective (complete search is presented in Multimedia Appendix 1). The search was performed on February 23, 2024.

### Screening

Title and abstracts were screened by three independent researchers (JW, CM, FB) using the Covidence (Veritas Health Innovation) program for systematic reviews. Two discussion sessions were organized to align the interpretation of the eligibility criteria. Two reviews were needed to reach a decision, and conflicts were resolved by discussion. Full-text screening was performed by JW, CM, and FB.

### Data Extraction

The data extraction form was reviewed and tested by three reviewers (CM, FB, JW). Data were extracted by CW, and JW verified all extraction data. The following data items were extracted per included study:

- Background information on the study: title, author, year of publication, country of origin, study design, and aim of the study
- Information on the telemedicine intervention: name of the intervention, purpose, type of telemedicine; users, and the moment of use within the neonatal care journey
- Methods of evaluation and the participants
- Outcomes of the study

**Figure 1.**
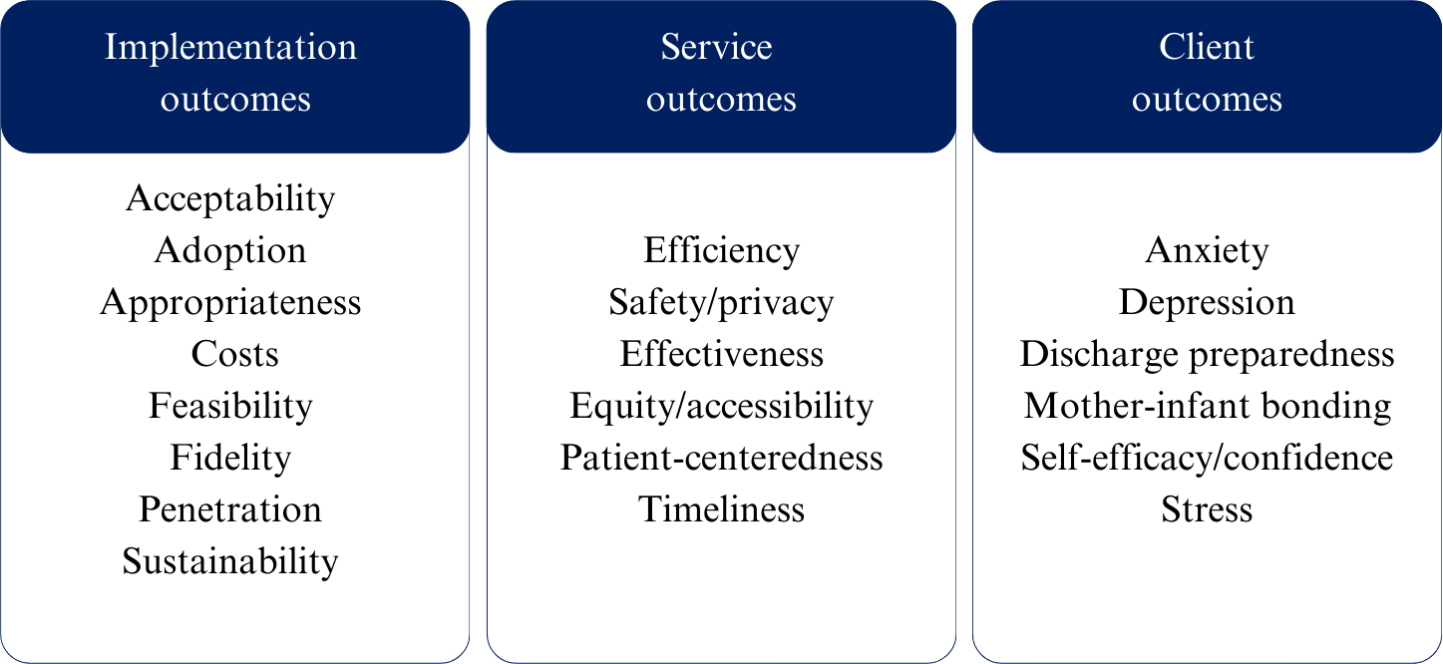
Outcome categorization used for this scoping review adapted from the framework of Proctor et al [25].

Outcomes were extracted following the implementation science framework by Proctor et al [25] suggesting the categorization of outcomes into implementation outcomes (how is the implementation used and appreciated in clinical practice?), service outcomes (how does the implementation influence the way clinical care is delivered?), and health outcomes (how does the implementation affect patient/parent outcomes?). Relevant outcomes for this specific scoping review are visualized in Figure 1. For health outcomes, this review focused on components of parental well-being, for instance, stress levels, confidence in their parenting role, and self-efficacy. This outcome framework categorizes PREMs into implementation or service outcomes and PROMs into health outcomes [21].

### Data Synthesis

Baseline characteristics of the included studies were presented for the study design, type of telemedicine, methods, and results categories. Extracted data were presented in an overview table and synthesized qualitatively for each step in the neonatal care journey. We defined the following steps in the journey: daily visits at the NICU, ongoing support at the NICU, transfers between NICUs, discharge from the hospital to home, and the first 12 months of follow-up.

### Ethics and Involvement

Due to the literature review nature of this study, ethical approval was deemed unnecessary. Parents and patients were involved in the design of the study, the interpretation of the results, and writing of the manuscript via experts from the neonatal parent and patient advocacy organization Care4Neo (author SOB).

## Results

### Characteristics of Included Studies

**Figure 2.**
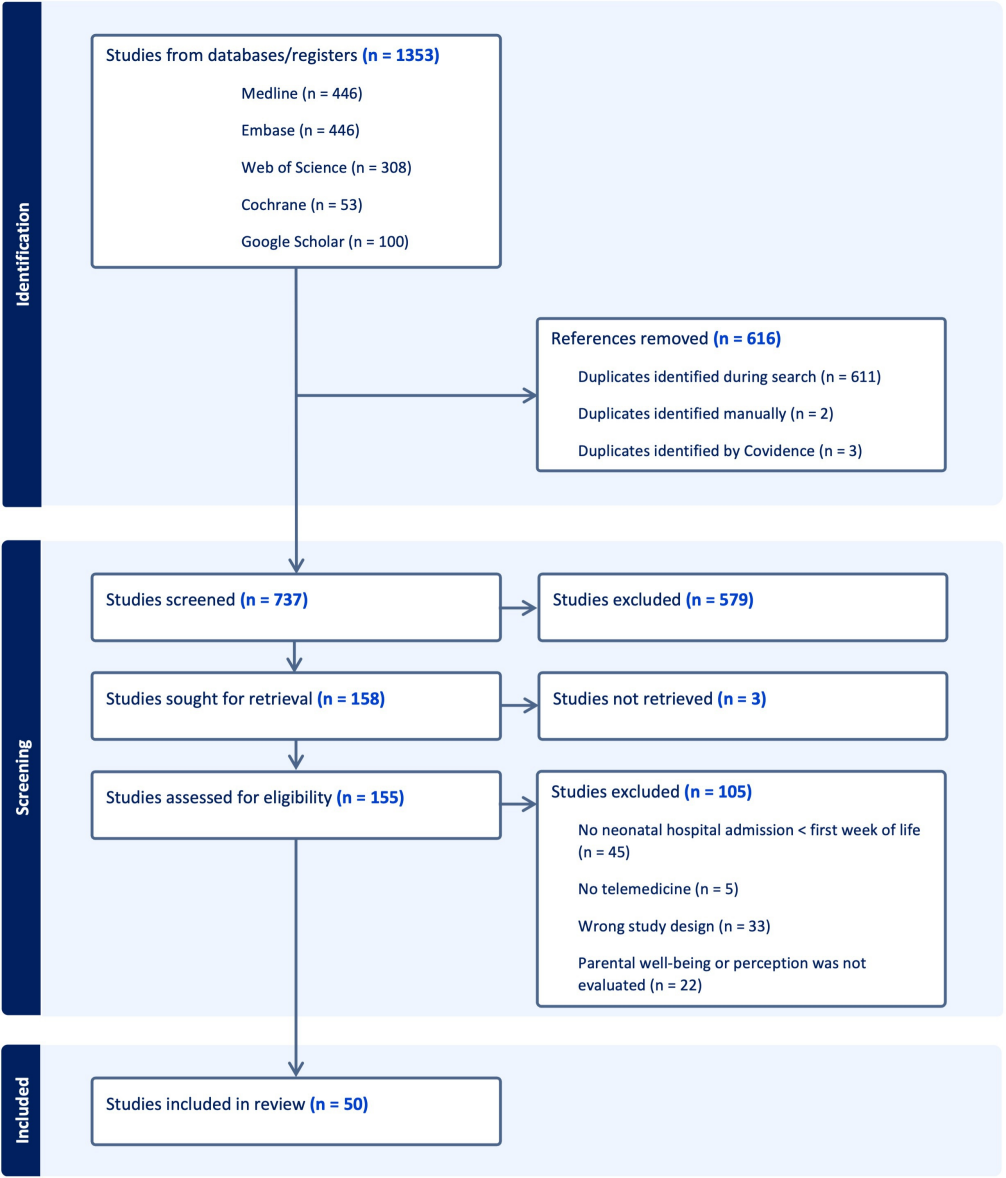
PRISMA (Preferred Reporting Items for Systematic Reviews and Meta-Analyses) inclusion flowchart.

After removing duplicates, 737 studies were selected for title and abstract screening. Interrater agreements were 78.9%, 82%, and 82.6% with a Cohen κ of 0.42, 0.59, and 0.48, respectively. Full texts were screened for 158 articles, resulting in the selection of 50 included studies (see flowchart in Figure 2). Complete data extraction of the included studies is presented in Multimedia Appendix 2. Included articles were predominantly published recently, with 76% (n=38) published in the last 5 years, and 50% (n=25) originated in the United States, with Scandinavian countries as the runner-up (n=10, 20%). Parents were included when designing the intervention in 12% (6/50) of studies. Frequently mentioned purposes of the telemedicine intervention were to remotely follow up on the neonate after discharge and save the family travel time (n=14, 28%) and to allow parents to see their infant at the NICU (n=11, 22%). All purposes are presented in Table 1.

Within the 50 articles, surveys (n=36, 72%) and interviews (n=10, 20%) were the most used evaluation methods, and they included 6 to 298 caregivers per study. Most of the interventions included real-time telemedicine technology (n=32, 64%) with 58% (29/50) including videoconferencing. Mobile apps facilitated the intervention in 21 studies, mainly used for educational or supporting content (8/21, 38%) and the transition to home and remote follow-up (15/21, 71%). Interventions were often part of a comprehensive health care program, using multiple technologies to facilitate different moments of contact between parents and health care providers (Multimedia Appendix 2).

**Table 1.** Purpose of the telemedicine interventions.

Purpose of telemedicine intervention	Frequency (n=50), n (%)
Remote follow-up and save the family travel time	14 (28)
Allow parents to see their infant in the NICU^a^	11 (22)
Improve transition from NICU to home	8 (16)
Provide psychosocial and emotional support	6 (12)
Provide education to enhance confidence and self-efficacy	5 (10)
Facilitate telerounds with a remote expert to prevent transfers	3 (6)
Provide regular medical updates to families	3 (6)

a NICU: neonatal intensive care unit.

### Outcome Measurements

The assessed outcome categories and methods of outcome measurement are presented in Table 2. Three out of 50 studies were in the design phase and were only able to hypothesize the outcomes, and therefore they were not included in the analysis of the outcome measures. The majority of studies used surveys (36/47, 77%) to assess the parents’ satisfaction and the effect on their well-being. Qualitative research methods such as interviews, focus groups, and workshops were applied in 23% (11/47) of studies and in all (3/3) studies designing an intervention. Of the 4 studies that used health records or usage logs, 2 studies presented usage logs in addition to qualitative data, whereas 2 studies only used data from the medical records system for establishing outcomes (Multimedia Appendix 2).

The surveys used to evaluate implementation outcomes varied. Some were validated questionnaires like the telemedicine usability questionnaire. Almost all used a 5-point Likert scale format. Service outcomes were either qualitative themes (eg, privacy concerns, accessibility of health care) or the potentially avoided travel distance or time. The Parental Stressor Scale (PSS-NICU) was the most frequently used standardized questionnaire (8/25, 32%) evaluating parental stress. The Parenting Sense of Competence Scale was used in 16% (4/25) of studies to evaluate self-efficacy. Nine of the 25 studies that evaluated client outcomes using surveys used a nonvalidated questionnaire, developed by the research team, to evaluate parental discharge preparedness, self-efficacy, or satisfaction with the delivered health care. An overview of the used questionnaires is shown in Multimedia Appendix 3. Client outcomes assessing the impact on the parents’ well-being were focused on depression, anxiety, stress, bonding, and self-efficacy.

**Table 2.** The number of studies assessing different outcome categories and the methods used.

Methods used	Implementation outcomes (n=33)^a^, n (%)	Service outcomes (n=11)^a^, n (%)	Client outcomes (n=31)^a^, n (%)
Surveys	26 (79)	8 (73)	25 (81)
Qualitative methods	9 (27)	3 (27)	7 (23)
Health records/usage logs	2 (6)	1 (9)	1 (3)

a Only 47 of the 50 articles were examined because the remaining 3 studies were in the design phase and were therefore only able to hypothesize the outcomes.

### Parental-Neonatal Care Journey

#### Overview of the Journey

**Figure 3.**
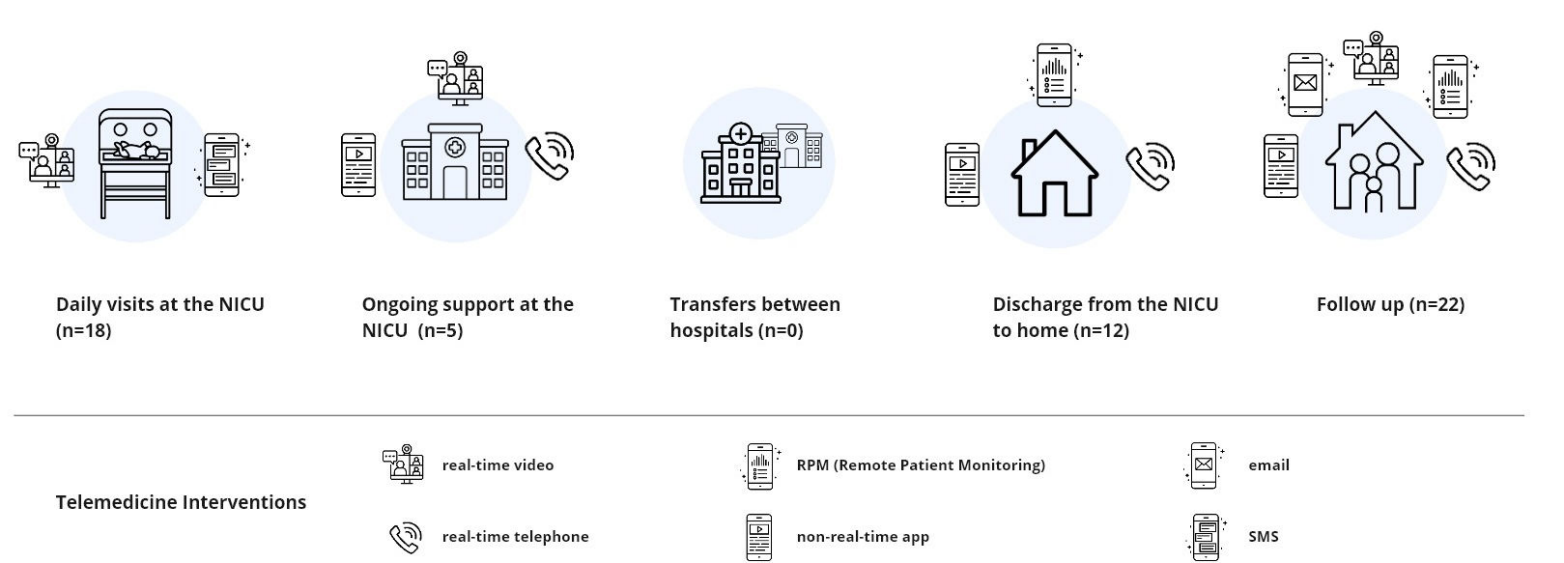
Overview of included telemedicine interventions for each step in the neonatal care journey. NICU: neonatal intensive care unit.

An overview of telemedicine interventions for each moment in the parental-neonatal care journey is presented in Figure 3. Their impact on implementation outcomes, service outcomes, and health outcomes is presented in Table 3. Most telemedicine interventions focused on the follow-up after hospital discharge (22/50, 44%), the discharge from the NICU to home (18/50, 36%), or daily visits to the NICU (18/50, 36%). None of the interventions focused on transfers between hospital wards or hospitals.

**Table 3.** Telemedicine interventions per moment in the parental-neonatal care journey.

Moment in the journey, purpose, and technology			Implementation outcomes	Service outcomes	Client outcomes
Daily visits at the NICU^a^ (n=18)					
Allow parents to see their infant remotely [26-36]					
Real-time video			*Acceptability*: high satisfaction *Adoption*: high usage rates *Appropriateness*: easy to use *Feasibility*: training of staff is necessary, technical issues *Sustainability*: how to schedule maintenance of the tool	Privacy concerns	Decreased stress (2 survey studies; PSS-NICU^b^) No difference in stress, anxiety, depression (2 survey studies; STAI-S^c^. PSS-NICU, MIB^d^, DASS-21^e^) Reduction of stress, anxiety and increase in confidence (3 interview studies) Increased stress due to video unavailability or traumatic images without explanation. Hypervigilance due to 24-7 video availability (interview study)
Telerounds with remote expert [37-39]					
Real-time video			*Acceptability*: high satisfaction. Comfortable talking to remote neonatologist *Feasibility*: high video/audio quality	Higher attendance of parents to daily rounds	Not evaluated^f^
Virtual family-centered rounds [36,40]					
Real-time video			*Acceptability*: high satisfaction *Adoption*: 50% used intervention *Appropriateness*: easy to use	Higher attendance of parents to daily rounds	NICU staff felt they had a positive impact on caregivers
Provide medical updates [41,42]					
Real-time video, SMS			Videoconferencing was *feasible*, *acceptable* and *reliable*	No impact on satisfaction with treatment and staff	No reduction in parental concerns (interview study)
Provide lactation support [43]					
Real-time video			Not evaluated	Not evaluated	Increased percentage exclusively breastfeeding
Ongoing support at the NICU (n=5)					
Educational, empower and reduce anxiety [44-48]					
Non–real-time app			*Acceptability*: satisfied with social support and interaction *Appropriateness*: perceived as useful	Not evaluated	Increased readiness for discharge Higher self-efficacy (survey study; PSOC^g^) No impact on stress and anxiety (survey study; PSS-NICU, GAD-7^h^). Hypothesized to serve as a source of emotional encouragement (one interview study)
Real-time telephone, video			*Acceptability*: high satisfaction *Fidelity*: 44% completed intervention	Not evaluated	Decreased anxiety and stress (survey study; BAI^i^, IDAS-GD^j^, EPDS^k^)
Transfers between hospitals (n=0)			Not evaluated	Not evaluated	Not evaluated
Discharge from the hospital to home (n=12)					
Educate and empower parents [48-54]					
Non–real-time app, RPM^l^, real-time telephone			*Acceptability*: high satisfaction *Appropriateness*: easy to use	Increases care accessibility	Increased discharge preparedness (survey study) Higher self-efficacy (survey study; PSOC) Increased mother-infant bonding (survey study; MPAS^m^) Decreased stress (survey study; PSS-NICU) No difference in parenting confidence (survey study; KPCS^n^, MABISC^o^)
Follow-up (n=22)					
Improve transition to home [51,52,54-58]					
Non–real-time app, RPM, real-time video			*Acceptability*: high satisfaction *Appropriateness*: easy to use *Feasibility*: high video/audio quality *Fidelity*: high rates of usage	Satisfied with discharge handoff. Replaced hospital visits thus saved travel time.	Earlier adjusting to home, establishing normalcy (interview study) Increased self-efficacy (survey study) Helped parents in their role (two studies)
Remote follow-up [50,59-72]					
Non–real-time app, RPM, emails, telephone			*Acceptability*: fun *Appropriateness*: easy to use *Feasibility*: remote scoring had equal quality as in-person *Fidelity*: high return rates of surveys	Not evaluated	Increased confidence (two interview studies) No increase in parenting confidence (survey study; KPCS, MABISC)
RPM app with real-time video			*Acceptability*: high satisfaction *Adoption*: 50% attendance to appointments *Appropriateness*: easy to use *Feasibility*: technical issues reported	Replaced hospital visits Improved approachability, affordability, and availability	Increased self-efficacy/confidence (3 survey studies; PSOC, one interview study)

a NICU: neonatal intensive care unit.

b PSS-NICU: Parental Stressor Scale–NICU.

c STAI-S: State-Trait Anxiety Inventory.

d MIB: Mother Infant Bonding Questionnaire.

e DASS-21: Depression Anxiety Stress Scale.

f Not evaluated was stated when none of the included studies were evaluated for the specific outcome measure.

g PSOC: Parenting Sense of Competence Scale.

h GAD-7: 7-item Generalized Anxiety Disorder.

i BAI: Beck Anxiety Inventory.

j IDAS-GD: Inventory of Depression and Anxiety Symptoms.

k EPDS: Edinburgh Postnatal Depression Scale.

l RPM: remote patient monitoring.

m MPAS: Maternal Postnatal Attachment Scale.

n KPCS: Karitane Parenting Confidence Scale.

o MABISC: Mother and Baby Interaction Scale.

#### Daily Visits to the NICU

All telemedicine interventions at the NICU used real-time audiovisual communication, either to allow parents to see their infants or for daily rounds with remote parents or experts. Facilitating a bedside camera accessible for parents resulted in high usage rates. It also resulted in a decrease in stress and anxiety, and an increase in confidence in multiple survey studies with randomized controlled designs [26,27] and interview studies [28-30]. Other studies found nonsignificant differences for stress, anxiety, and bonding with evident positive trends in open-ended questions [31,32].

Three important negative outcomes were found: technical aspects, privacy, and hypervigilance. Incidence of technical issues ranged widely (from 5% to 60%) and staff reported doubts about training and maintenance protocols [27,28,31,41]. Furthermore, unexpected unavailability of the video connection triggered stress and fear in parents, not knowing what was happening to their infant [28,33]. Privacy concerns for both the infant and the NICU staff were mentioned [28,33]. Extensive security evaluation of the data connection prior to implementation was included in most study protocols to ensure privacy for the infant. Privacy of the NICU staff, on the other hand, was an unexpected negative outcome in two studies [27,33]. Their main concerns were the fear of behaving differently when being watched by parents while taking care of the infant and the liability risk for neonatologists when medical emergencies are being recorded. Lastly, the possibility of watching your infant 24-7 resulted in hypervigilance for some parents [28]. Parents explained that being able to constantly watch their infant made their home feel less restful. Virtual family-centered rounds, some with remote experts present, all had high satisfaction rates. Rosenthal et al [40] showed a relatively low adoption rate, with only 48.6% of parents attending the virtual rounds at least once. However, there was an evident increase in parental participation during rounds in this group, with the attendance rate being 3.4 times higher compared to the control group without virtual rounds, suggesting a positive effect in a selected group of parents. Makkar et al [37] also found higher participation rates in the telerounds with remote experts. Impact on parental health outcomes was not evaluated for virtual rounds.

#### Ongoing Support and Education

Although interventions for educational and supportive purposes targeted different moments in the neonatal care journey, they shared the same telemedicine aspects. These interventions were designed as comprehensive programs including a smartphone- or tablet-based application with interactive functions and planned moments of contact with health care professionals.

An example is the “My Bridgham Baby” app [44], including (1) practical information regarding the NICU; (2) information on the role as a parent for their admitted infant; (3) support services for parents and their families; (4) discharge education, checklists and milestones before discharge; (5) mental health services for parents; (6) advice on financial and insurance resources; and (7) information regarding the follow-up. This app also includes a chat function to ask questions to the medical team. Self-efficacy [48,59] and discharge preparedness increased [44,49], and one controlled trial showed nonsignificant trends in parenting confidence [50]. Two studies included parental perspectives in the design phase by hosting focus groups, both recommending the integration of social support facilitation into the app [45,54]. Interventions that included real-time telemedicine (telephone or video) showed a decrease in anxiety and stress [46,47,53].

#### Discharge Preparedness and Follow-Up

Almost half of the included studies focused on the transition to home and follow-up (24/50, 48%). The majority of these interventions (16/24, 67%) used a combination of telemedicine modalities: apps and videoconferences. Only a few of them (3/24, 13%) used the app as a means to actively gather information on the patient [57,61,66], the definition of remote patient monitoring. As expected, studies concluded that telemedicine results in accessible health care, saving travel time and money for parents [51,55,56,58,68,72]. Furthermore, the parental sense of competence at home increased, probably due to the easily accessible option of asking questions to professionals [52,55,58,61,63,65,66,71,72].

## Discussion

### Principal Findings

In this scoping review, we aimed to identify the potential of telemedicine interventions to improve parental well-being during the neonatal care journey of their infant. Telemedicine interventions included in our review had two main purposes: (1) to overcome physical distance through remote follow-ups and virtual family-centered rounds with remote experts, allowing parents to see their infant remotely, and (2) to prepare parents for discharge by providing information and social support. The majority of the interventions were part of a comprehensive program, entailing a telemedicine intervention with, for instance, a structured set-up over time, multiple functionalities, and moments of contact with health care providers. Of the included studies, 70% (33/47) evaluated parental opinions on the intervention, like usability and technical quality, and 66% (31/47) assessed the actual impact of the intervention on the parents’ well-being, like stress, self-efficacy, depression symptoms, and unmet needs. This review draws attention to three main gaps in the literature:

- None of the telemedicine interventions focused on the transfers between hospitals, despite this being an urgent unmet need for parents [11].
- There is a need for assessing not only implementation satisfaction but also the actual impact on the parents’ well-being, ideally in both the short- and long-term.
- The variety of methods and questionnaires used to evaluate parents’ well-being and experiences creates a great challenge to compare the outcomes of different studies, which is a commonly mentioned issue when integrating results of parents’ well-being evaluations at the NICU [9]. Also, the variety of methods for evaluating telemedicine interventions aligns with a recent systematic review examining the evaluation of patient and staff experience with remote patient monitoring, which similarly noted a lack of consensus and standardization in evaluation methods [73].

Categorization of telemedicine interventions was challenging. Included interventions were often part of a comprehensive program including multiple technologies. Unfortunately, a significant number of included studies failed to fully describe the program and, perhaps more importantly, to present the results behind parents’ satisfaction with the intervention. Moreover, the majority of the underlying studies provided limited information on the characteristics of the included parents. Factors such as age, socioeconomic status, family situation, and ethnicity influence parental needs [74,75] and are relevant when interpreting the observed impact of telemedicine, individualizing interventions, and ensuring availability of technology to all patients. Integrating findings from the included studies on both intervention satisfaction and its impact on parents’ well-being was therefore challenging. To enhance generalizability and implementation, it is recommended to provide comprehensive descriptions of the entire program and the included participants, and to particularly highlight intervention components that are vital for parents.

Furthermore, the impact on parental well-being is mainly established in studies using technology as a means to provide emotional or educational support. This suggests using the technology should not be the primary goal, but that the potential impact derived from the actual content of the delivered care, education, or support should be the goal. In other words, the telemedicine intervention should be the means and not the end. On the other hand, usage and uptake of the telemedicine intervention are essential in order to reach the intended impact. It remains important to reflect on the parental needs that are being targeted by the intervention and if telemedicine is the best intervention to improve the experience. More comprehensive, probably qualitative, research into the parental journey and the unmet needs is advised before designing new interventions. Subsequently, it is essential to invite parents to participate when designing and implementing a telemedicine intervention, using a participatory study design with, for instance, co-creation sessions.

### Strengths and Limitations

By purposefully including the whole range of technological interventions, including mature technologies like telephone consultations, SMS, or email services which have been part of health care for decades [76], we aimed to create a comprehensive overview. Furthermore, by including studies that evaluate not only the impact on parental well-being but also parental perceptions of telemedicine interventions, we were able to demonstrate the current level of parental involvement in the different phases of telemedicine research, including designing, implementing, and evaluating interventions. Also, we did not limit our inclusion to specific study designs. These three considerations resulted in a complete overview of all technological interventions that have been studied. Despite the scoping nature of the review, and therefore missing the quality appraisal, the three reviewers facilitated a robust and transparent inclusion process by predefining eligibility criteria, performing validation sessions, and extracting data with two independent researchers.

Despite efforts to be comprehensive, this review likely missed some studies. More specifically, by excluding protocols and trial registrations, we overlooked ongoing studies, such as a cluster randomized controlled trial for virtual family-centered hospital rounds [77] and the neoPARTNER study [78]. Considering the majority of included studies were published within the last 5 years, we expect the number of studies currently being performed to be relevant. Another challenge in this review was to capture the entire scope of the parental journey, as a lack of universal terminology and definitions posed significant obstacles. To minimize the risk of missing important parental aspects, we chose very broad terms for the search string (experience, perspective, perception, depression, anxiety, stress, satisfaction, etc). With this extensive search string, we screened and included articles focusing also on parents’ views of the technology instead of solely reviewing the impact on their care journey.

### Practical Implications

Based on the included studies, several practical recommendations can be provided. This review clearly found educational and supportive telemedicine interventions, often delivered through a mobile or tablet application with multiple functionalities, have a positive impact on discharge preparedness. Furthermore, bedside cameras can be useful to improve infant bonding and reduce stress when caregivers are unable to be present at the NICU. However, an important remark is that privacy concerns of medical personnel, hypervigilance, and increased stress are pitfalls of the continuous availability of a bedside camera. Remote follow-up was often provided by a telemedicine program that includes an app and scheduled contact moments with health care providers (video or telephone). While remote follow-up improves the accessibility of health care by saving parents travel time and making parental confidence increase, it has not been proven to reduce stress, depression, or anxiety. The opportunities of telemedicine interventions, as described above, prove to be effective when used in the context of a comprehensive telemedicine program including informational provisions, moments of communication, and social support.

### Conclusion

Telemedicine interventions have the opportunity to improve parents’ well-being during their neonatal care journey, especially when enhancing discharge preparedness and when aiming to overcome physical distance using bedside webcams, virtual family-centered rounds, or remote follow-ups. We advise future researchers to (1) properly describe their telemedicine intervention to enhance generalizability and (2) assess the impact on parents’ well-being when evaluating the intervention, ideally using a combination of validated questionnaires (PROMs; eg, the Parental Stressor Scale) and in-depth interviews. Furthermore, when designing and piloting new interventions, a critical reflection on the targeted parental needs, by involving parents in the study and using co-creation sessions, is essential to improve their journey.

## Supplementary material

10.2196/60610 Multimedia Appendix 1 Search strategy.

10.2196/60610 Multimedia Appendix 2 Data extraction overview.

10.2196/60610 Multimedia Appendix 3 Used questionnaires for measuring parent-reported outcome measures and parent-reported experience measures.

10.2196/60610 Checklist 1 PRISMA-ScR (Preferred Reporting Items for Systematic Reviews and Meta-Analyses Extension for Scoping Reviews) checklist.

## Abbreviations

NICU: neonatal intensive care unit
PICO: Population, Intervention, Comparator, Outcome
PREM: patient/parent-reported experiences measure
PRISMA-ScR: Preferred Reporting Items for Systematic Reviews and Meta-Analyses Extension for Scoping Reviews
PROM: patient/parent-reported outcome measure
