# Supplementary material for: Opportunities for Telemedicine to Improve Parents’ Well-Being During the Neonatal Care Journey: Scoping Review
Source: JMIR Pediatr Parent. 2024 Dec 2;7:e60610. doi: 10.2196/60610 (PMC11627525; doi:10.2196/60610)
Supplement: Multimedia Appendix 1 [file pediatrics-v7-e60610-s001.docx]

**SUPPLEMENTAL SEARCH STRATEGY**

| **Database searched** | **Platform** | **Years of coverage** | **Records** | **Records after duplicates removed** |
| --- | --- | --- | --- | --- |
| Medline ALL | Ovid | 1946 - Present | 446 | 442 |
| Embase | Embase.com | 1971 - Present | 446 | 155 |
| Web of Science Core Collection* | Web of Knowledge | 1975 - Present | 308 | 72 |
| Cochrane Central Register of Controlled Trials | Wiley | 1992 - Present | 53 | 17 |
| Additional Search Engines: Google Scholar** (100 top-ranked) | | | 100 | 56 |
| **Total** | | | **1353** | **742** |

*Science Citation Index Expanded (1975-present) ; Social Sciences Citation Index (1975-present) ; Arts & Humanities Citation Index (1975-present) ; Conference Proceedings Citation Index- Science (1990-present) ; Conference Proceedings Citation Index- Social Science & Humanities (1990-present) ; Emerging Sources Citation Index (2005-present)

**Google Scholar was searched via "Publish or Perish" to download the results in EndNote.

No other database limits were used than those specified in the search strategies

Search strategy:

Exclude conference abstracts and Cochrane registrations. Only English language.

**Embase 446**

('telemedicine'/de OR 'telediagnosis'/de OR 'teleconsultation'/de OR 'telesurgery'/de OR 'telecare'/exp OR 'telehealth'/exp OR 'telemonitoring'/exp OR 'telecommunication'/mj/de OR 'digital health'/exp/mj OR 'digital health technology'/exp OR 'digital health intervention'/exp OR 'electronic consultation'/exp OR 'mhealth'/exp OR 'mobile health'/exp/mj OR 'videoconferencing'/de OR (telehospital* OR tele-hospital* OR telehealth* OR tele-health* OR telemedic* OR tele-medic* OR telemonitor* OR tele-monitor* OR telecare OR tele-care OR tele-icu* OR tele-intensive-car* OR telepresence* OR tele-presence* OR tele-referral* OR telereferral* OR teleconsultat* OR teleradiol* OR tele-radiol* OR ((remot* OR electronic* OR virtual*) NEAR/3 (health* OR intervention* OR consult* OR diagno* OR medicine*)) OR ((remot*) NEAR/3 (monitor*)) OR ((smart*) NEAR/3 (health*)) OR uhealth OR ((digital* OR remot* OR electronic* OR virtual* OR mobile) NEAR/3 (distanc*) NEAR/3 (practice*)) OR videoconferenc* OR video-conferenc* OR smartwatch* OR smart-watch* OR teleNICU OR tele-NICU OR tele-neo* OR teleneo*):ab,ti,kw OR (e-health* OR ehealth* OR mhealth* OR m-health* OR ((digital*) NEAR/3 (health* OR intervention* OR consult* OR diagno* OR medicine*)) OR mobile-health* OR remote-consultat*):ti) **AND** ('neonatal intensive care unit'/de OR newborn/de OR prematurity/exp OR 'newborn care'/exp OR (((neonat* OR neo-nat* OR newborn*) NEAR/3 (intensive-care* OR IC OR ICU)) OR neonate* OR neo-nate* OR newborn* OR (premature NOT premature-death*) OR pre-mature OR baby OR babies OR preterm* OR pre-term* OR NICU OR NICUs OR teleNICU OR teleneonat*):ab,ti,kw) **AND** (parent/exp OR caregiver/de OR 'formal caregiver'/de OR (parent OR parents OR parental OR father* OR mother* OR caregiver* OR care-giver*):ab,ti,kw) **AND** (experience/de OR anxiety/exp OR perspective/de OR 'mental stress'/exp OR depression/exp OR 'patient attitude'/exp OR trust/de OR 'family attitude'/exp OR satisfaction/exp OR 'evaluation study'/exp OR (experience* OR anxiet* OR perspective OR stress* OR feeling* OR willing* OR depress* OR attitude OR acceptance* OR acceptabil* OR perception* OR satisfaction* OR satisfied OR participation* OR engagement OR engaging* OR evaluation* OR ((parent*) NEAR/3 (outcome*)) OR trust*):ab,ti,kw) NOT ([Conference Abstract]/lim OR [Conference Review]/lim) AND [ENGLISH]/lim

**Medline 446**

(exp "Telemedicine"/ OR *"Telecommunications"/ OR "Videoconferencing"/ OR (telehospital* OR tele-hospital* OR telehealth* OR tele-health* OR telemedic* OR tele-medic* OR telemonitor* OR tele-monitor* OR telecare OR tele-care OR tele-icu* OR tele-intensive-car* OR telepresence* OR tele-presence* OR tele-referral* OR telereferral* OR teleconsultat* OR teleradiol* OR tele-radiol* OR ((remot* OR electronic* OR virtual*) ADJ3 (health* OR intervention* OR consult* OR diagno* OR medicine*)) OR ((remot*) ADJ3 (monitor*)) OR ((smart*) ADJ3 (health*)) OR uhealth OR ((digital* OR remot* OR electronic* OR virtual* OR mobile) ADJ3 (distanc*) ADJ3 (practice*)) OR videoconferenc* OR video-conferenc* OR smartwatch* OR smart-watch* OR teleNICU OR tele-NICU OR tele-neo* OR teleneo*).ab,ti,kf. OR (e-health* OR ehealth* OR mhealth* OR m-health* OR ((digital*) ADJ3 (health* OR intervention* OR consult* OR diagno* OR medicine*)) OR mobile-health* OR remote-consultat*).ti.) **AND** (Intensive Care Units, Neonatal/ OR exp Infant, Newborn/ OR Intensive Care, Neonatal/ OR (((neonat* OR neo-nat* OR newborn*) ADJ3 (intensive-care* OR IC OR ICU)) OR neonate* OR neo-nate* OR newborn* OR (premature NOT premature-death*) OR pre-mature OR baby OR babies OR preterm* OR pre-term* OR NICU OR NICUs OR teleNICU OR teleneonat*).ab,ti,kf.) **AND** (exp Parents/ OR Caregivers/ OR (parent OR parents OR parental OR father* OR mother* OR caregiver* OR care-giver*).ab,ti,kf.) **AND** (exp Anxiety/ OR exp Stress, Psychological/ OR Depression/ OR exp Psychological Distress/ OR exp Attitude/ OR Trust/ OR exp Patient Satisfaction/ OR Personal Satisfaction/ OR Evaluation Study/ OR (experience* OR anxiet* OR perspective OR stress* OR feeling* OR willing* OR depress* OR attitude OR acceptance* OR acceptabil* OR perception* OR satisfaction* OR satisfied OR participation* OR engagement OR engaging* OR evaluation* OR ((parent*) ADJ3 (outcome*)) OR trust*).ab,ti,kf.) NOT (congres* OR abstract*).pt. AND english.la.

**Cochrane 53**

((telehospital* OR tele-hospital* OR telehealth* OR tele-health* OR telemedic* OR tele-medic* OR telemonitor* OR tele-monitor* OR telecare OR tele-care OR tele-icu* OR tele-intensive-car* OR telepresence* OR tele-presence* OR tele-referral* OR telereferral* OR teleconsultat* OR teleradiol* OR tele-radiol* OR ((remot* OR electronic* OR virtual*) NEAR/3 (health* OR intervention* OR consult* OR diagno* OR medicine*)) OR ((remot*) NEAR/3 (monitor*)) OR ((smart*) NEAR/3 (health*)) OR uhealth OR ((digital* OR remot* OR electronic* OR virtual* OR mobile) NEAR/3 (distanc*) NEAR/3 (practice*)) OR videoconferenc* OR video-conferenc* OR smartwatch* OR smart-watch* OR teleNICU OR tele-NICU OR tele-neo* OR teleneo*):ab,ti OR (e-health* OR ehealth* OR mhealth* OR m-health* OR ((digital*) NEAR/3 (health* OR intervention* OR consult* OR diagno* OR medicine*)) OR mobile-health* OR remote-consultat*):ti) **AND** ((((neonat* OR neo-nat* OR newborn*) NEAR/3 (intensive-care* OR IC OR ICU)) OR neonate* OR neo-nate* OR newborn* OR (premature NOT premature-death*) OR pre-mature OR baby OR babies OR preterm* OR pre-term* OR NICU OR NICUs OR teleNICU OR teleneonat*):ab,ti) **AND** ((parent OR parents OR parental OR father* OR mother* OR caregiver* OR care-giver*):ab,ti) **AND** ((experience* OR anxiet* OR perspective OR stress* OR feeling* OR willing* OR depress* OR attitude OR acceptance* OR acceptabil* OR perception* OR satisfaction* OR satisfied OR participation* OR engagement OR engaging* OR evaluation* OR ((parent*) NEAR/3 (outcome*)) OR trust*):ab,ti) NOT ("conference abstract":kw OR Trial registry record:pt)

**Web of Science 308**

(TS=(telehospital* OR tele-hospital* OR telehealth* OR tele-health* OR telemedic* OR tele-medic* OR telemonitor* OR tele-monitor* OR telecare OR tele-care OR tele-icu* OR tele-intensive-car* OR telepresence* OR tele-presence* OR tele-referral* OR telereferral* OR teleconsultat* OR teleradiol* OR tele-radiol* OR ((remot* OR electronic* OR virtual*) NEAR/2 (health* OR intervention* OR consult* OR diagno* OR medicine*)) OR ((remot*) NEAR/2 (monitor*)) OR ((smart*) NEAR/2 (health*)) OR uhealth OR ((digital* OR remot* OR electronic* OR virtual* OR mobile) NEAR/2 (distanc*) NEAR/2 (practice*)) OR videoconferenc* OR video-conferenc* OR smartwatch* OR smart-watch* OR teleNICU OR tele-NICU OR tele-neo* OR teleneo*) OR TI=( e-health* OR ehealth* OR mhealth* OR m-health* OR ((digital*) NEAR/2 (health* OR intervention* OR consult* OR diagno* OR medicine*)) OR mobile-health* OR remote-consultat*)) **AND** TS=(((((neonat* OR neo-nat* OR newborn*) NEAR/2 (intensive-care* OR IC OR ICU)) OR neonate* OR neo-nate* OR newborn* OR (premature NOT premature-death*) OR pre-mature OR baby OR babies OR preterm* OR pre-term* OR NICU OR NICUs OR teleNICU OR teleneonat*)) **AND** ((parent OR parents OR parental OR father* OR mother* OR caregiver* OR care-giver*)) **AND** ((experience* OR anxiet* OR perspective OR stress* OR feeling* OR willing* OR depress* OR attitude OR acceptance* OR acceptabil* OR perception* OR satisfaction* OR satisfied OR participation* OR engagement OR engaging* OR evaluation* OR ((parent*) NEAR/2 (outcome*)) OR trust*))) NOT DT=(Meeting Abstract OR Meeting Summary) AND

LA=(English)

**Google Scholar**

telemedicine|telehealth|telemonitor|telecare|ehealth neonate|neonatal|newborn|preterm|NICU|NICUs|teleNICU "parent|parents|parental|father|mother|caregiver experience|perspective|stress|attitude|acceptance|acceptability|perception|satisfaction|evaluation"

telemedicine|telehealth|telemonitor|telecare|ehealth neonate|neonatal|newborn|preterm|NICU|NICUs|teleNICU 'parent|parents|parental|father|mother|caregiver experience|perspective|stress|attitude|acceptance|acceptability|perception|satisfaction|evaluation'
