# Supplementary material for: Opportunities for Telemedicine to Improve Parents’ Well-Being During the Neonatal Care Journey: Scoping Review
Source: JMIR Pediatr Parent. 2024 Dec 2;7:e60610. doi: 10.2196/60610 (PMC11627525; doi:10.2196/60610)
Supplement: Multimedia Appendix 3 [file pediatrics-v7-e60610-s003.docx]

Table 1. Used questionnaires to evaluate impact on parent's wellbeing

| **Questionnaire** | **Parents’ wellbeing theme(s)** | **Frequency of use (n studies)** |
| --- | --- | --- |
| Alabama Parenting Questionnaire | Parenting skills | 1 |
| Beck Anxiety Inventory | Anxiety | 1 |
| Depression Anxiety Stress Scale (DASS) | Depression, anxiety, stress | 1 |
| Edinburgh Postnatal Depression Scale | Depression | 1 |
| Generalized Anxiety Disorder -7 | Anxiety | 1 |
| Hospital Anxiety and Depression Scale (HADS) | Anxiety, depression | 1 |
| Inventory of Depression and Anxiety Symptoms (IDAS-GD) | Depression, anxiety | 1 |
| Karitane Parenting Confidence Scale | Self-efficacy | 1 |
| Maternal Postnatal Attachment Scale | Infant bonding | 1 |
| Mother and Baby Interaction Scale | Infant bonding | 1 |
| Mother Infant Bonding Questionnaire (MIB) | Infant bonding | 1 |
| Parenting Sense of Competence Scale (PSOC) | Self-efficacy | 4 |
| Parenting Stress Index–Short Form | Stress | 1 |
| Parental Stressor Scale (PSS-NICU, PSS-Infant hospitalization) | Stress | 8 |
| Pediatric Quality of Life Inventory (PedsQL) Healthcare Satisfaction Generic Module | Healthcare satisfaction | 1 |
| Penticuff Parent Understanding Survey | Parenting skills | 1 |
| Readiness for Hospital Discharge Scale | Discharge preparedness | 1 |
| State-Trait Anxiety Inventory (STAI) | Anxiety | 1 |
| Non-validated questionnaires developed by the research team | Discharge preparedness, self-efficacy, satisfaction with healthcare | 9 |
